# Apolipoprotein E deficiency accelerates atherosclerosis development in miniature pigs

**DOI:** 10.1242/dmm.036632

**Published:** 2018-10-10

**Authors:** Bin Fang, Xueyang Ren, Ying Wang, Ze Li, Lihua Zhao, Manling Zhang, Chu Li, Zhengwei Zhang, Lei Chen, Xiaoxue Li, Jiying Liu, Qiang Xiong, Lining Zhang, Yong Jin, Xiaorui Liu, Lin Li, Hong Wei, Haiyuan Yang, Rongfeng Li, Yifan Dai

**Affiliations:** 1 Jiangsu Key Laboratory of Xenotransplantation, Nanjing Medical University, Nanjing 211166, China; 2 Key Laboratory of Targeted Intervention of Cardiovascular Disease, Collaborative Innovation Center for Cardiovascular Disease Translational Medicine, Nanjing Medical University, Nanjing 211166, China; 3 Huai'an First Hospital Affiliated to Nanjing Medical University, Department of Pathology, Huai'an 223300, China; 4 Department of Laboratory Animal Science, College of Basic Medicine, Army Medical University, Chongqing 400038, China; 5 State Key Laboratory of Reproductive Medicine, Nanjing Medical University, Nanjing 211166, China; 6 Shenzhen Xenotransplantation Medical Engineering Research and Development Center, Institute of Translational Medicine, Shenzhen Second People's Hospital, First Affiliated Hospital of Shenzhen University, Shenzhen 518035, China

**Keywords:** Atherosclerosis, *ApoE*, Bama miniature pigs, CRISPR/Cas9

## Abstract

Miniature pigs have advantages over rodents in modeling atherosclerosis because their cardiovascular system and physiology are similar to that of humans. Apolipoprotein E (ApoE) deficiency has long been implicated in cardiovascular disease in humans. To establish an improved large animal model of familial hypercholesterolemia and atherosclerosis, the clustered regularly interspaced short palindromic repeats (CRISPR)-associated protein 9 system (CRISPR/Cas9) was used to disrupt the *ApoE* gene in Bama miniature pigs. Biallelic-modified *ApoE* pigs with in-frame mutations (*ApoE^m/m^*) and frameshift mutations (*ApoE^−/−^*) were simultaneously produced. *ApoE^−/−^* pigs exhibited moderately increased plasma cholesterol levels when fed with a regular chow diet, but displayed severe hypercholesterolemia and spontaneously developed human-like atherosclerotic lesions in the aorta and coronary arteries after feeding on a high-fat and high-cholesterol (HFHC) diet for 6 months. Thus, these *ApoE^−/−^* pigs could be valuable large animal models for providing further insight into translational studies of atherosclerosis.

## Abstract (precis)

**Editor's choice:** *ApoE* knockout pigs displayed severe hypercholesterolemia and spontaneously developed human-like atherosclerotic lesions in the aorta and coronary arteries within 6 months of feeding on a high-fat and high-cholesterol diet.

## INTRODUCTION

Atherosclerosis is a chronic inflammatory disease characterized by the accumulation of proliferative smooth muscle cells, macrophages, lipids, cholesterol, calcium deposits and cellular debris in arterial walls (Libby et al., 2011; Mastenbroek et al., 2015). The most common clinical manifestations of this disease are myocardial infarction, ischemic stroke and ischemic damage to kidneys and intestines, due to occlusive thrombosis and severe arterial stenosis (Libby et al., 2013; Murray et al., 2012). Although the pathogenesis of atherosclerosis remains elusive, plasma lipid levels, particularly those of low-density lipoprotein (LDL) cholesterol, correlate with the risk of atherogenesis.

Apolipoprotein E (ApoE) is a major component of very-low-density lipoprotein (VLDL) and chylomicrons, playing a critical role in the metabolism of cholesterol and clearance of lipoprotein remnants (Mahley, 1988). ApoE was initially recognized for its importance in lipoprotein metabolism and cardiovascular disease because dysfunction of ApoE protein results in familial dysbetalipoproteinemia (type III hyperlipoproteinemia), which often leads to atherosclerosis (Eichner et al., 2002; Georgiadou et al., 2011; Ghiselli et al., 1981). Thus, *ApoE* knockout (KO) rodent models have been favored in atherosclerosis studies and have provided valuable insights into mechanisms underlying this disease (Chistiakov et al., 2017; Iqbal et al., 2016; Niimi et al., 2016; Piedrahita et al., 1992; Plump et al., 1992). However, rodent models have limitations because many important human atherosclerosis features, including plaque rupture and thrombosis, rarely occur in rodents (Al-Mashhadi et al., 2013; Bentzon and Falk, 2010). In addition, rodents are dissimilar to humans in vascular size and lipoprotein profiles and metabolism, which have limited studies on intravascular devices and hampered the translation of experimental findings into clinic applications (Al-Mashhadi et al., 2013; Langheinrich et al., 2007). Hence, suitable large animal models that can adequately mimic human atherosclerosis are needed.

Currently, pigs are broadly used as large animal models in biomedical research because they share many similarities with humans with respect to physiology, pathology, immunology and the cardiovascular system (Aigner et al., 2010; Reyes et al., 2014; Xi et al., 2004). Moreover, compared to other large animals such as non-human primates, pigs are more ethically and economically acceptable, making them preferable models for cardiovascular disease research. However, it is very difficult to develop advanced atherosclerotic lesions in normal pigs, even with a high-fat and high-cholesterol (HFHC)-diet induction. These considerations suggest the need for the development of genetically modified miniature pigs.

The clustered regularly interspaced short palindromic repeats (CRISPR)-associated protein 9 (CRISPR/Cas9) system has emerged as a powerful and efficient tool for gene editing (Hsu et al., 2014; Mali et al., 2013; Niu et al., 2014), and has significantly facilitated the generation of genetically modified pig models (Chen et al., 2015; Estrada et al., 2015; Hai et al., 2014; Zhang et al., 2017). Recently, there was a report of *ApoE*/LDL receptor (*LDLR*) double-KO minipigs produced by CRISPR/Cas9-mediated gene targeting (Huang et al., 2017). Although slightly increased plasma lipid levels were observed, an atherosclerosis phenotype was not ever described in those pigs, possibly because in-frame (IF) mutations in both the *ApoE* and *LDLR* alleles may give rise to truncated ApoE and LDLR proteins with partially retained function (Huang et al., 2017).

In the present study, we used the CRISPR/Cas9 system to disrupt the *ApoE* gene, and established *ApoE* KO Bama miniature pigs by somatic cell nuclear transfer (SCNT) technology. We have found that *ApoE* KO pigs fed an HFHC diet had severe hypercholesterolemia and spontaneously developed human-like atherosclerotic lesions in the aorta and coronary arteries. These results indicated that the *ApoE* KO pigs are an ideal model for the study of human atherosclerosis.

## RESULTS

### sgRNA-directed site-specific indels in primary pig fetal fibroblasts (PFFs)

**Fig. 1.**
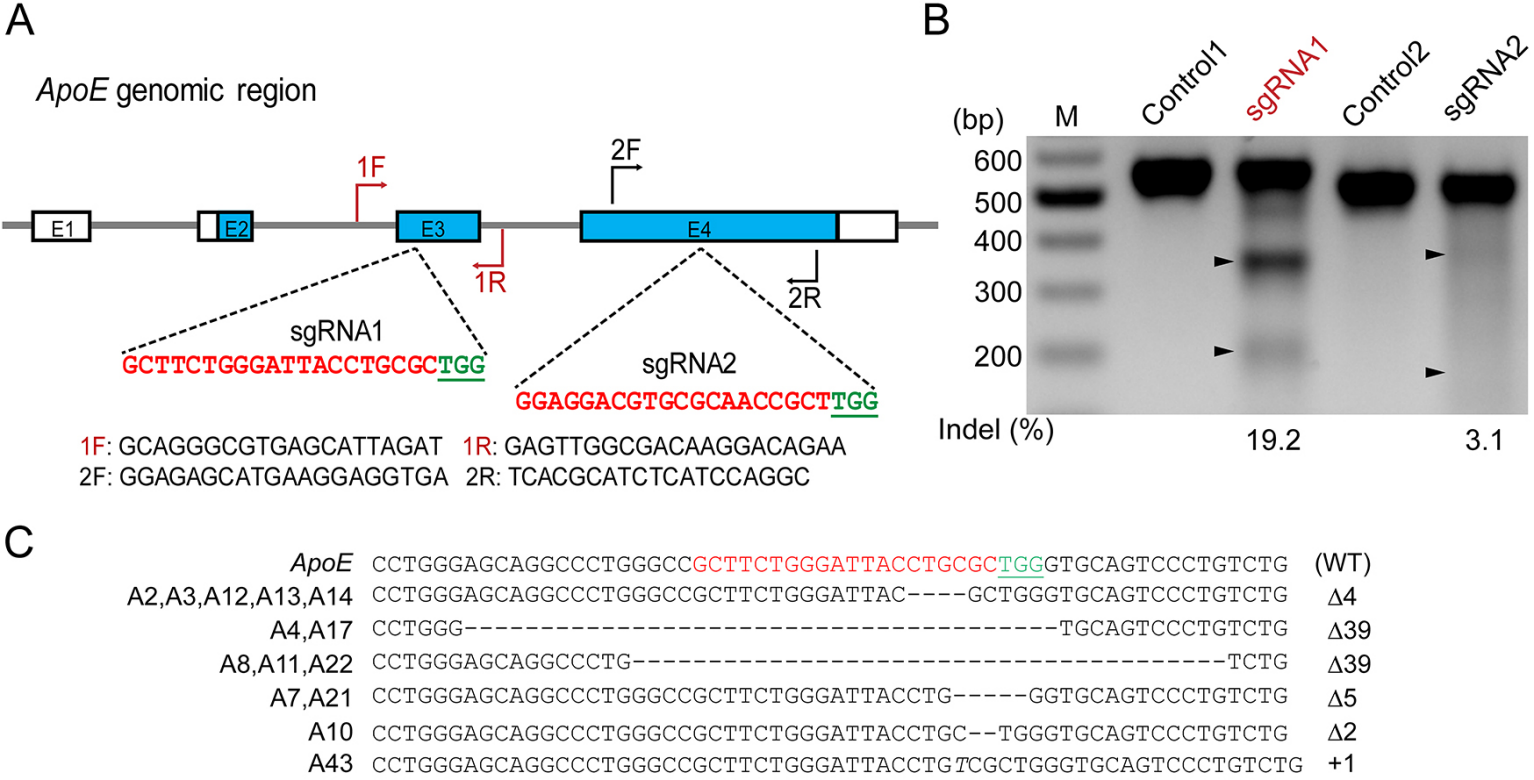
**CRISPR/Cas9 mediates *ApoE* gene targeting in PFFs.** (A) Schematic diagram of *Cas9*-sgRNA targeting sites of the pig *ApoE* locus. The sgRNA targeting sequences are shown in red, and the protospacer-adjacent motif (PAM) sequences are shown in green and underlined. (B) T7E1 assay for Cas9-mediated cleavage at *ApoE* targeting sites in PFFs. M: DNA marker; Controls: PCR products of untransfected PFFs treated with T7E1; sgRNA1 and sgRNA2: PCR products of PFFs transfected with Cas9-sgRNA1 and Cas9-sgRNA2 treated with T7E1, respectively. (C) Genotypes of homozygous *ApoE* biallelic-modified colonies. The WT sequence is shown at the top. Deletion (Δ); insertion (+); italic letter denotes the inserted base pair.

To disrupt the *ApoE* gene in Bama miniature pigs, two single-guide RNAs (sgRNAs) were designed based on exon 2 and 3 sequences determined by PCR sequencing. The precise target sites are shown in Fig. 1A. To test their cleavage efficiency, these sgRNAs were cloned into a Cas9 targeting vector, pX330 (#42230, Addgene, Cambridge, MA, USA) and introduced into PFFs. At 48 h post-transfection, primary PFFs were harvested for genomic DNA extraction. PCR amplicons spanning the *ApoE* target region were subjected to a T7 endonuclease 1 digestion assay followed by gel electrophoresis. The cleavage fragments shown in Fig. 1B indicated that both sgRNAs could induce insertions/deletions (indels) in the target region of the *ApoE* gene, although sgRNA1 (19.2%) had higher cleavage efficiency than sgRNA2 (3.1%). To establish *ApoE* KO cell lines, the Cas9-sgRNA1 targeting vector was co-transfected with a neomycin expression plasmid into early passages of PFFs derived from a 35-day-old male Bama mini fetus. After 10 days of selection with G418, resistant cell colonies were harvested. PCR amplification was performed on each colony and the resultant fragments were subjected to TA cloning for Sanger sequencing. Of the 44 colonies analyzed, 27 were identified as having biallelic modifications, 14 of which were homozygous, with 6 indel types ranging from 1 to 39 base pairs (bp) in the *ApoE* gene target region (Fig. 1C).

### Production of *ApoE* KO pigs by SCNT

**Fig. 2.**
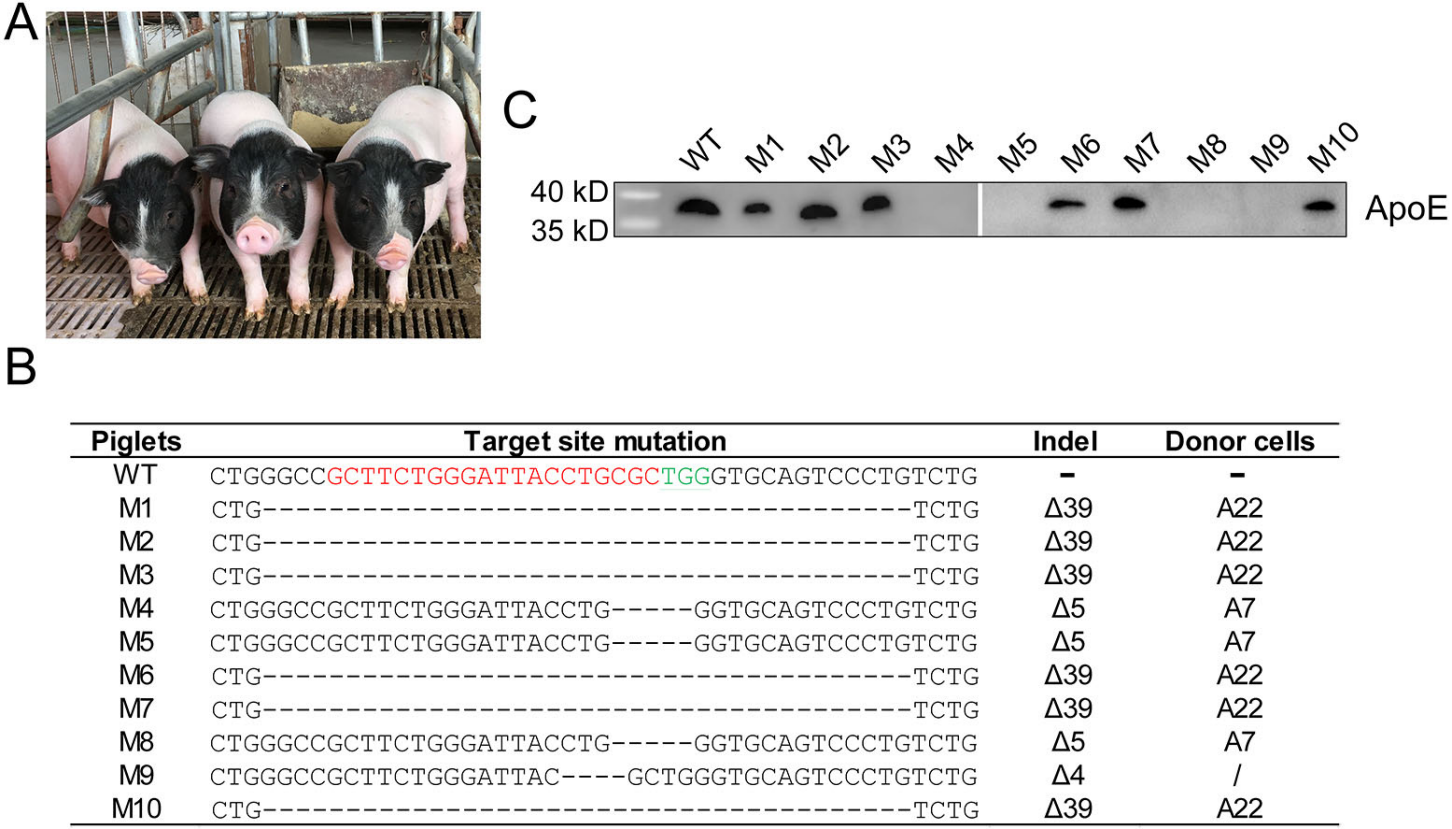
**Generation of *ApoE*-gene-targeted piglets via SCNT.** (A) Litter of *ApoE* targeted male founder piglets. (B) Genotypes of cloned piglets. The WT sequence is shown at the top, in which the sgRNA sequences are shown in red, and the PAM sequences are shown in green. Deletion (Δ). The genotype of piglet M9 was different from A7 and A22 donor cells. (C) Representative western blot of plasma ApoE protein of cloned piglets and WT control.

*ApoE* biallelic-modified colonies (A7 and A22) were pooled and used as nuclear donors for SCNT to produce *ApoE* KO piglets. Morula- and blastula-stage reconstructed embryos were transferred to 7 surrogates, yielding 3 full-term pregnancies, from which 17 live-born male piglets were delivered (Fig. 2A). Seven of them died before weaning (Table 1). Genomic DNA from the ear tissues of the 10 remaining piglets (designated M1 to M10) was isolated, and mutations were detected via PCR and Sanger sequencing. Piglets M1, M2, M3, M6, M7 and M10 carried a 39 bp deletion corresponding to A22 donor cells; piglets M4, M5 and M8 harbored a 5 bp deletion mutation derived from A7 donor cells; and piglet M9 bore a 4 bp deletion in the *ApoE* target region that did not originate from the A7 and A22 colonies, indicating that a small portion of cells with a 4 bp deletion in the *ApoE* region was in the donor-cell mixture (Fig. 2B).

**Table 1.**
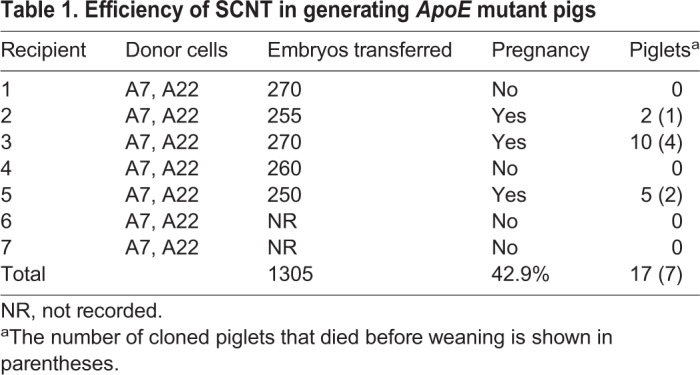
**Efficiency of SCNT in generating *ApoE* mutant pigs**

Because the undesired off-target effects of the CRISPR/Cas9 system are still a concern, we computationally predicted off-target sites by screening the pig genome using an online tool (http://www.rgenome.net/cas-offinder/) based on sequence homology to the *ApoE* sgRNA1 sequence, allowing for un-gapped alignments with up to 4 mismatches. In this way, a total of 81 potential off-target sites (OTSs) were identified. To verify whether off-target cleavage occurred in these genetically modified piglets, 25 OTSs (Table S1) were randomly selected for PCR amplification and subjected to Sanger sequencing. The primers for amplifying the off-target sites are listed in Table S2. The results indicated that none of the sequencing reads exhibited indel mutations, suggesting that there were no off-target effects at these sites.

### Elevated serum cholesterol levels in *ApoE*^−/−^ pigs

**Fig. 3.**
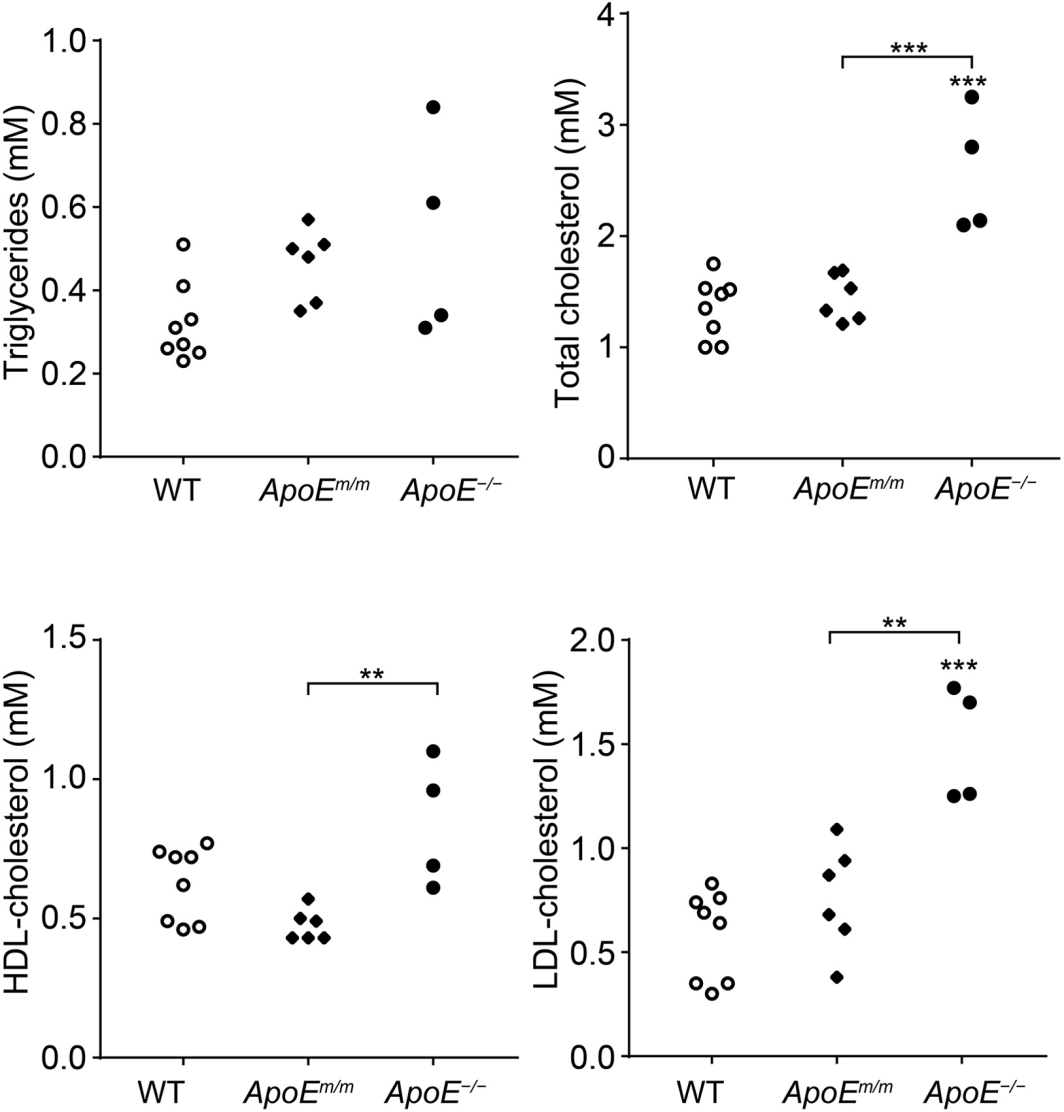
**Plasma triglycerides and cholesterol of *ApoE* targeted piglets and WT controls fed a low-fat diet.** Data represent fasting plasma measurements at 8 weeks of age in *ApoE^m/m^* piglets (*n*=6), *ApoE^−/−^* piglets (*n*=4) and age-matched WT pigs (*n*=8). **P<0.01, ***P<0.0001 versus the control group (ANOVA). All values represent the means±s.d.

Body weights and appetites of the *ApoE* targeted piglets, and their age-matched wild-type (WT) Bama minipigs, were comparable at birth and after weaning (data not shown). Plasma ApoE in all piglets was determined by western blot analysis. The ApoE protein was absent in mutant piglets harboring a 4 or 5 bp deletion (M4, M5, M8 and M9), whereas the mutant piglets bearing a 39 bp deletion showed obvious ApoE expression (Fig. 2C). We speculated that a 4 or 5 bp deletion in the *ApoE* gene region would give rise to a frameshift (FS) mutation, whereas a 39 bp deletion would lead to a 13 amino acids (aa) IF deletion in the ApoE protein. To assess the potential effects of different *ApoE* gene mutations on serum lipids, these 10 mutant piglets were categorized into *ApoE^m/m^* (13 aa deletion) and *ApoE*^−/−^ (FS mutation) groups. Plasma cholesterol and triglyceride levels were measured in mutant and WT animals 4 weeks after weaning. No significant difference in fasting serum triglyceride concentrations was observed among WT, *ApoE^m/m^* and *ApoE*^−/−^ piglets. The *ApoE^m/m^* founders had comparable total cholesterol level as the controls. However, a significantly increased serum cholesterol was observed in *ApoE*^−/−^ piglets [2.57±0.55 mM (mean±s.d.)] compared to WT (1.35±0.27 mM) and *ApoE^m/m^* pigs (1.45±0.21 mM), which was mostly contributed by LDL cholesterol (Fig. 3).

### Severe hypercholesterolemia during HFHC feeding

**Fig. 4.**
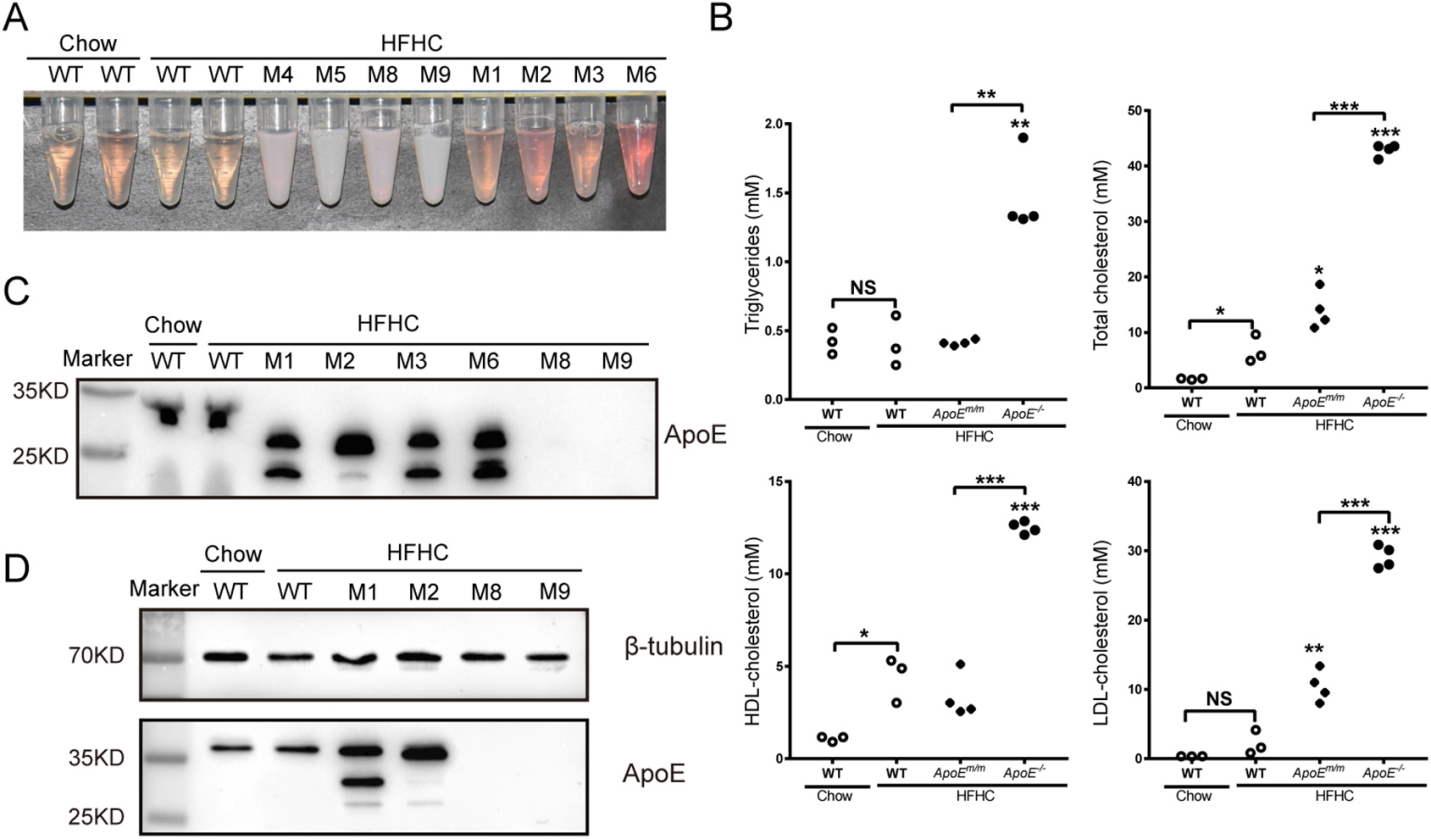
**Phenotype of *ApoE* targeted piglets during HFHC feeding.** (A) Representative image of fasting serum of *ApoE* targeted piglets fed a HFHC diet, and age-matched WT animals fed a HFHC diet and normal chow for 3 months. *ApoE^−/−^* piglet serum had a turbid appearance. (B) Plasma triglycerides and cholesterol of *ApoE* targeted and WT piglets fed a HFHC diet for 3 months (NS, not significant; *P<0.05, **P<0.01, ***P<0.0001 versus the control group, ANOVA). HFHC-fed WT pigs were also compared with chow-fed WT pigs (Student's *t*-test). All values represent the means±s.d. (C) Representative western blot of plasma ApoE protein of cloned piglets and WT control after 6 months feeding of HFHC diet. (D) Representative western blot of liver ApoE protein of cloned piglets and WT control after 6 months feeding with a HFHC diet.

Three-month-old *ApoE* targeted piglets and age-matched WT pigs were challenged with a HFHC diet containing 30% saturated fat and 1.5% cholesterol for 3 consecutive months. WT pigs fed with chow diet were used as controls. Fasting blood was collected monthly for the assessment of plasma cholesterol and triglyceride levels. During this period, two *ApoE^m/m^* piglets (M7 and M10) died because of infection. Unlike WT and *ApoE^m/m^* piglets, *ApoE*^−/−^ piglets maintained on a HFHC diet exhibited chylemia, as indicated by the turbid appearance of their sera (Fig. 4A). Compared to WT pigs on normal chow, increased total cholesterol levels, mostly contributed by high-density lipoprotein (HDL) cholesterol, were observed in WT pigs fed an HFHC diet. However, triglyceride levels did not significantly differ between WT pigs fed an HFHC or normal diet (Fig. 4B). Among all groups, the *ApoE*^−/−^ group had the highest levels of triglycerides and total cholesterol after 3 months of HFHC feeding, with mean levels of 1.47 and 42.86 mM, respectively (Fig. 4B). *ApoE^m/m^* piglets showed less severe hypercholesterolemia, as their total cholesterol levels were about 2-fold that of WT groups on HFHC feeding (14.96 vs 6.76 mM), and their plasma triglyceride levels were comparable to that of WT controls (0.52 vs 0.40 mM) (Fig. 4B). LDL but not HDL cholesterol levels were significantly increased in HFHC-fed *ApoE^m/m^* pigs compared to HFHC-fed WT pigs. Interestingly, both LDL and HDL cholesterol levels were dramatically elevated in the HFHC-fed *ApoE*^−/−^ group compared to the other groups.

### Progressive atherosclerosis in *ApoE* targeted pigs

**Fig. 5.**
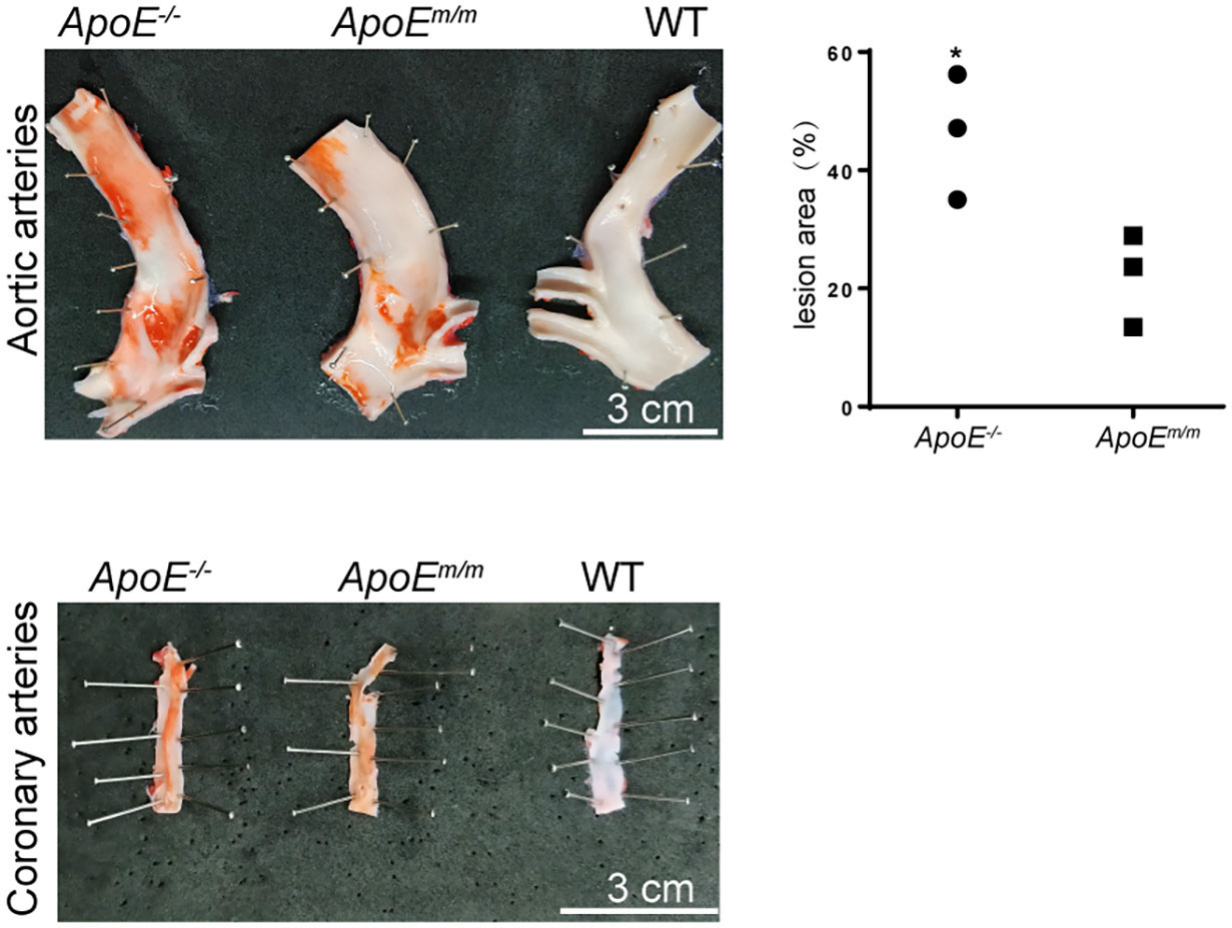
**Aortic and coronary atherosclerosis in *ApoE* targeted piglets fed a HFHC diet.** Left: photographs of Sudan-IV-stained aorta (top) and coronary arteries (bottom) of *ApoE^m/m^* and *ApoE^−/−^* pigs showed atherosclerotic lesions. Right: the percentage of Sudan-IV-stained area was measured by ImageJ software. *P<0.05.

**Fig. 6.**
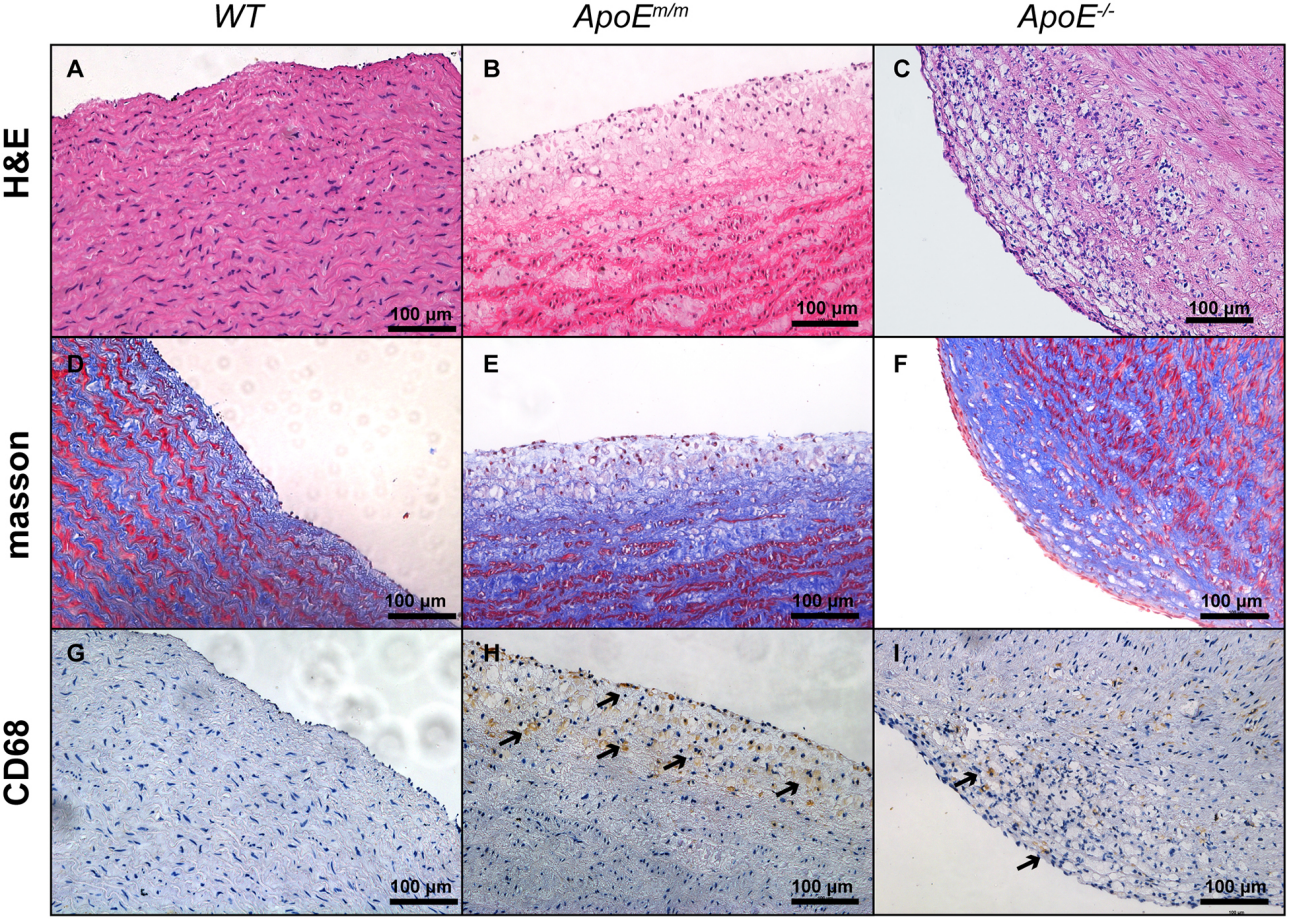
**Representative histological images of aorta from WT, *ApoE^m/m^* and *ApoE^−/−^* pigs.** No visible atherosclerosis was seen in the aorta from WT pigs (A,D,G). *ApoE^m/m^* pigs had pathological fatty streak of the aorta (B,E,H). *ApoE^−/−^* pigs showed progressive atherosclerotic lesions in the luminal part of the arterial intima (C,F,I).

To facilitate development of atherosclerosis plaques in these pigs, HFHC diet feeding was prolonged to 6 months. Serum ApoE protein in the WT, *ApoE^m/m^* and *ApoE*^−/−^ groups was examined by western blot. As expected, ApoE protein was absent in *ApoE*^−/−^ piglets. An upregulated expression of ApoE was observed in *ApoE^m/m^* piglets compared to WT controls (Fig. 4C). Moreover, the molecular mass of ApoE protein in *ApoE^m/m^* piglets was smaller than that in WT animals, due to the deletion of 13 aa in the ApoE protein or degradation of the truncated ApoE protein. To test whether disruption of ApoE accelerated atherosclerosis development, 2 pigs from each group (WT, *ApoE^m/m^* and *ApoE^−/−^*) were euthanized after 6 months of HFHC feeding. ApoE expression in these sacrificed pig livers was also examined by western blot with β-tubulin as an internal control. Consistent with serum ApoE levels, ApoE expression was elevated in the *ApoE^m/m^* group and absent in the *ApoE*^−/−^ group compared to WT pigs (Fig. 4D). The *ApoE*^−/−^ pigs displayed a significantly higher percentage of aortic surface area covered with lesions than *ApoE^m/m^* pigs, namely a 2.1-fold increase compared with the *ApoE^m/m^* pigs (Fig. 5). However, atherosclerotic lesions were barely seen in the aorta and coronary arteries of WT pigs even when fed a HFHC diet. Interestingly, coronary area covered with atherosclerotic lesion was comparable between *ApoE^m/m^* and *ApoE^−/−^* pigs fed a HFHC diet (Fig. 5). The aorta from WT, *ApoE^m/m^* and *ApoE^−/−^* pigs were further stained to assess the presence of atherosclerosis. WT pigs on HFHC feeding exhibited no significant pathological alterations, whereas *ApoE^m/m^* pigs had appreciable aorta areas of fatty streaks, intimal thickening as well as apparent foam cell accumulation as indicated by CD68 staining. The *ApoE^−/−^* pigs showed progressive atherosclerotic lesions of fibrous plaque, which was symbolic of fibrosis as well as hyaline degeneration in aorta (Fig. 6).

## DISCUSSION

Pigs have advantages over rodent models in recapitulating the human pathophysiology of atherosclerosis because they are similar to humans with regard to vascular size and physiology. Modeling atherosclerosis in minipigs has been hampered by a low propensity to atherosclerosis even with long-term feeding with atherogenic diets. Genetically modified pig models have been considered promising in solving the problem. However, utilization of genetically modified pigs as atherosclerosis models is still in its infancy, with the exception of PSCK9 transgenic pigs created by transposon integration and *LDLR* KO pigs generated by homology-directed recombination (HDR)-based methods (Al-Mashhadi et al., 2013; Davis et al., 2014; Li et al., 2016). Here, we successfully established novel porcine models of accelerated atherosclerosis in Bama miniature pigs by disrupting the endogenous *ApoE* gene using the CRISPR/Cas9 system. These pigs exhibited moderate, yet consistently, elevated serum cholesterol levels when fed a standard chow diet, and showed severe hypercholesterolemia and developed aortic and coronary atherosclerotic lesions when fed a HFHC diet.

With high mutagenesis efficiency, CRISPR/Cas9 technology has been extensively used for gene targeting in pigs (Chen et al., 2015; Estrada et al., 2015; Zhang et al., 2017). Consistent with previous studies, high gene-targeting efficiency of the CRISPR/Cas9 system was achieved in PFFs: 11 and 28 of 48 colonies were identified as monoallelic and biallelic *ApoE* modified, respectively, which was much higher than that garnered with HDR-based *ApoE* targeting in rodents (Piedrahita et al., 1992; Plump et al., 1992). A total of 17 homozygous *ApoE* KO piglets were produced by a single round of SCNT and no off-target mutagenesis was detected in them, supporting that the high efficiency and relative simplicity of the CRISPR/Cas9 technology can greatly facilitate the efficient production of gene KO pigs. Due to its preference for the error-prone non-homologous end joining (NHEJ) pathway, the CRISPR/Cas9 system has the advantage of being able to induce multiple gene modifications. Founder pigs with −4 or −5 bp deletions in the *ApoE* targeting site were generated and showed identical serum lipid profiles. Thus, we suspected that epigenetic status had little impact on the phenotype of the cloned founder pigs.

Hypercholesterolemia has long been implicated in atherosclerosis. Similar to *PCSK9* transgenic pigs and *LDLR* KO pigs, the *ApoE^−/−^* pigs showed increased plasma cholesterol levels on a low-fat diet, resembling the human familial hypercholesterolemia phenotype. Interestingly, when fed a HFHC diet for 3 months, total plasma cholesterol levels plateaued to ∼40 mM in *ApoE^−/−^* pigs, but only reached ∼20-25 mM in *PCSK9* transgenic and *LDLR* KO pigs. In addition, HDL cholesterol levels were dramatically increased in *ApoE^−/−^* pigs, whereas the *PCSK9* transgene and *LDLR* KO did not exert statistically significant effects on HDL cholesterol levels as compared to WT controls. Contrary to relatively low triglyceride levels in *PCSK9* and *LDLR* KO pigs, hypertriglyceridemia was observed in *ApoE^−/−^* pigs by HFHC feeding. Thus, the *ApoE^−/−^* pigs could be beneficial for delineating the underlying pathophysiology of hypercholesterolemia and atherosclerosis because their serum lipid profiles differ from existing pig models, which cannot sufficiently recapitulate human disease. More recently, a line of *ApoE*/*LDLR* KO pigs generated by CRISPR/Cas9-mediated gene targeting has been reported (Huang et al., 2017). Surprisingly, cholesterol levels in those *ApoE*/*LDLR* KO pigs were lower than those of *LDLR* KO pigs reported previously (Davis et al., 2014; Li et al., 2016) or *ApoE* KO pigs in this study. Moreover, ApoE protein was still present in the *ApoE*/*LDLR* KO pigs and no atherosclerosis phenotype was reported. Considering that those pigs harbored a −18 bp deletion in the *ApoE* gene and +1/−15 bp indels in the *LDLR* gene, we speculated that IF-mutated ApoE and LDLR proteins did not completely lose their functions. Thus, the *ApoE^−/−^* pigs established in this study are the first *ApoE* KO pig model with severe atherosclerosis phenotypes.

Although the results presented here are encouraging, there were some limitations to this study. First, we did not investigate the advanced stages of atherosclerosis, such as plaque rupture or thrombosis. Thus, a study with a longer duration is needed to establish the full spectrum of atherosclerotic plaque progression from onset to rupture. Second, this study also did not take into account the effects of gender and puberty on phenotype because only male *ApoE^−/−^* pigs were investigated and development of coronary atherosclerosis was observed in the *ApoE^−/−^* pigs on HFHC feeding for 6 months, when they have reached puberty. Finally, in addition to its implication in lipoprotein metabolism and cardiovascular disease, ApoE exerts pleiotropic roles in Alzheimer's disease (Shi et al., 2017; Yu et al., 2014), immunoregulation (Braesch-Andersen et al., 2013) and cognition (Davies et al., 2014). However, the developmental consequences of ApoE depletion in other tissues/organs were not evaluated, being beyond the scope of this study.

In conclusion, *ApoE^−/−^* pigs fed a HFHC diet showed severe hypercholesterolemia and developed progressive atherosclerotic lesions. These pigs could serve as ideal large animal models for the elucidation of *ApoE* gene functions and translational studies of atherosclerosis.

## MATERIALS AND METHODS

### Ethics statement

WT mature Landrace gilts and WT Bama minipigs were purchased from Chia Tai Co., Ltd (Huaiyin, China) and housed in a large animal facility affiliated with Nanjing Medical University, Nanjing, China. Standard chow diets were purchased from Chia Tai Co., Ltd and the HFHC diet was purchased from Xietong Organism Co., Ltd (Nanjing, China). Standard pig husbandry procedures were applied to all animals. Embryo transplantation and sacrifice procedures were conducted under isoflurane anesthesia to minimize animal suffering. All animal experiments were carried out in accordance with the guidelines approved by the Institutional Animal Care and Use Committee (IACUC) of the Nanjing Medical University.

### CRISPR/Cas9 plasmid constructs

To target the porcine *ApoE* gene, sgRNAs were designed using online tools (http://crispr.mit.edu/). The DNA oligos of sgRNAs were purchased from Genscript (Nanjing, China) (*ApoE* sgRNA1: 5′-CACCGCTTCTGGGATTACCTGCGC-3′, 5′-AAACGCGCAGGTAATCCCAGAAGC-3′; and *ApoE* sgRNA2: 5′-CACCGGAGGACGTGCGCAACCGCT-3′, 5′-AAACAGCGGTTGCGCACGTCCTCC-3′). These complementary DNA oligos were annealed and ligated into *Bbs*I (Thermo Fisher Scientific, Waltham, MA, USA)-digested pX330 (Addgene plasmid 423230) using thermal conditions 37°C for 30 min, 95°C for 5 min, and annealed by decreasing by 5°C/min to 25°C to generate the *Cas9*-sgRNA targeting plasmids for *ApoE*.

### T7E1 cleavage assay

PFFs were isolated from Chinese Landrace fetuses at day 35 of gestation using 200 U/ml collagenase (Invitrogen) and 25 kU/ml DNaseI (Invitrogen), and cultured as previously described (Chen et al., 2015). PFFs transfected with or without *Cas9*-sgRNA targeting plasmids (as described above) were cultured for 48 h. Genomic DNA was extracted using a DNA extraction kit (TianGen, Beijing, China) and the genomic regions surrounding the CRISPR/Cas9 target site were PCR amplified. PCR primers used are as follows: 5′-CCTCAGGTGGTCTAGGTTGG-3′ and 5′-TTTTGCAATGGAGGAGTGGC-3′ for sgRNA1; 5′-CGCCTCTCTCTGTTCATTGC-3′ and 5′-TTTTGCAATGGAGGAGTGGC-3′ for sgRNA2. The PCR conditions were as follows: 95°C for 5 min, followed by 30 cycles of 95°C for 10 s, 60°C for 30 s, and 72°C for 40 s, and a final 72°C for 7 min. A total of 250 ng of the purified PCR product was mixed with NEB Buffer 2, denatured, and annealed to allow formation of heteroduplexes using the following conditions: 95°C for 5 min, 95°C to 85°C ramping at −2°C/s, 85°C to 25°C at −0.1°C/s, and 4°C hold. After reannealing, the products were digested with 1 μl of T7 endonuclease I (NEB, Beverly, MA, USA) at 37°C for 15 min and then run on a 2% agarose gel stained with ethidium bromide.

### PFF transfection and selection

Approximately 5 μg of the *ApoE Cas9*-sgRNA targeting plasmid was co-transfected with 1 μg of the neomycin expression plasmid (pCMV-tdTomato) into 1×10^6^ early passage of PFFs using the Basic Fibroblast Nucleofection Kit (Amaxa Biosystems/Lonza, Cologne, Germany), according to the manufacturer's instructions. A total of 800 μg/ml of G418 (Gibco, Grand Island, NY, USA) was applied to the post-transfection cells 48 h later and maintained for 10-14 days thereafter. The G418-resistant colonies were seeded in 24-well plates and then passaged to 12-well plates. Approximately 1/5 of the cells of a single colony were lysed with NP-40 (55°C for 30 min, 95°C for 10 min) for PCR screening and 4/5 of the remaining cells were used for SCNT. The primers used in amplifying the target regions were as follows: forward: 5′-CCTCAGGTGGTCTAGGTTGG-3′, reverse: 5′-TTTTGCAATGGAGGAGTGGC-3′. The PCR conditions were as follows: 95°C for 5 min, followed by 35 cycles of 95°C for 10 s, 60°C for 30 s, and 72°C for 50 s, and a final 72°C for 4 min. The PCR products were subcloned into a pMD18-T vector (Takara Clontech, Tokyo, Japan) according to the manufacturer's instructions. Fifteen to 20 individual clones were picked and sequenced.

### SCNT and embryo transfer

The oocytes for SCNT were collected from ovaries purchased from a local slaughterhouse and cultured for 42-44 h *in vitro*. The mature oocytes were enucleated as described elsewhere (Li et al., 2017). A single targeted PFF cell was transferred into the perivitelline space of the enucleated oocyte. After electrofusion of membranes between the donor cell and recipient cytoplast, the reconstructed embryos were cultured in embryo development medium at 38.5°C for 24 h. About 250 reconstructed oocytes were transferred into the uterus of an estrus-synchronized recipient gilt. Pregnancy was confirmed by ultrasound 30 days after transplantation and monitored until the perinatal period. All cloned piglets were born by normal delivery.

### Serum lipid chemistry

Whole blood was drawn from the jugular veins of overnight-fasted pigs at the indicated time points into serum-separating tubes (BD Diagnostics, Franklin Lakes, NJ, USA). Sera were prepared by centrifugation at 500 rcf for 15 min at room temperature. The levels of total cholesterol, triglycerides, HDL cholesterol and LDL cholesterol were determined by Dian Diagnostics (Nanjing, China).

### Western blot analysis

Western blotting was performed with 10-fold diluted plasma samples or 20-50 μg of liver tissue extract. Proteins were separated on 15% SDS-PAGE gels and transferred to polyvinylidene fluoride membranes (Bio-Rad, Hercules, CA, USA). The membranes were incubated overnight at 4°C with anti-ApoE primary antibody (1:2000; Novus Biological, Littleton, CO, USA). The membranes were incubated with goat anti-rabbit IgG horseradish peroxidase (HRP)-conjugated secondary antibody (1:2000; CWBiotech, Shanghai, China) and proteins were detected with the SuperSignal West Pico Chemiluminescent Substrate (Thermo Fisher Scientific) with a ChemiDoc Touch Imaging System (Bio-Rad). β-tubulin was used as an internal control with anti β-tubulin primary antibody (1:10,000; Proteintech, Rosemont, IL, USA) and goat anti-mouse HRP-conjugated secondary antibody (1:2000; Santa Cruz Biotechnology, Santa Cruz, CA, USA).

### Histology analysis

The aorta and coronary arteries were isolated from overnight-fasted pigs and fixed in 10% buffered formalin. Sudan IV was used to stain the atherosclerotic lesions. Images were collected on an Olympus FSX100 microscope (Olympus, Tokyo, Japan). The stained area was quantified using ImageJ software (NIH, Bethesda, MD, USA) and expressed as a percentage of the total image area. The aorta was divided into 1-cm segments taken perpendicular to the direction of blood flow and embedded in paraffin. H&E, Masson's trichrome and immunohistochemistry staining were conducted on 5 μm sections to confirm lesion details as described previously (Chen et al., 2018). CD68 monoclonal antibody (KP1; 1:100; Thermo Fisher Scientific) and HRP-conjugated goat anti-mouse secondary antibody (1:500; Proteintech, Rosemont, IL, USA) were used for immunostaining.

### Statistical methods

All data are expressed as the means±s.d. *P*-values were determined using a 2-tailed Student's *t*-test or 1-way analysis of variance (ANOVA) with a Tukey's *post hoc* correction for multiple group comparisons. A *P*-value of less than 0.05 was considered statistically significant.

## Supplementary Material

Supplementary information
