# Supplementary material for: Apolipoprotein E deficiency accelerates atherosclerosis development in miniature pigs
Source: Dis Model Mech. 2018 Oct 10;11(10):dmm036632. doi: 10.1242/dmm.036632 (PMC6215431; doi:10.1242/dmm.036632)
Supplement: Supplementary information [file dmm-11-036632-s1.pdf]

Table S1: Prediction of off-target sites of *ApoE*-sgRNA. Mismatched sites are in lowercase and highlighted in red.

	Coordinates	Targets	PAM
ApoE-sgRNA	chr6: 47270894-47270916	GCTTCTGGGATTACCTGCGC	TGG
OTS1	chr6:156975239-156975217	GCTTCTGGGATTcCCTGCtC	GGG
OTS2	chr10:33205421-33205399	GagTCTGGGATTACCTGgGC	AGG
OTS3	chr6:65545313-65545335	cCTaCTcGGAcTACCTGCGC	TGG
OTS4	chr2:60022835-60022813	GgTTCTGcGATTACCaGCGC	GGG
OTS5	chr14:142578303-142578325	GCTgCTGGGATTcCCTaCGC	AGG
OTS6	chr11:81262051-81262029	GCTcCTGGGggaACCTGCGC	AGG
OTS7	chr8:571425-571447	tCTTCTGGGtTcACCTGCGt	CGG
OTS8	chr12:49961319-49961341	cCTcCTGGGATgcCCTGCGC	AGG
OTS9	chr7:41278039-41278017	GagTCTGGGATgACCTGCtC	TGG
OTS10	chr7:128016676-128016654	GCTgCTaGcATTtCCTGCGC	AGG
OTS11	chr3:3625560-3625538	GCTgCTGGGcTcACCTGCaC	TGG
OTS12	chr13:186007669-186007647	GaTTCTtGGAgTgCCTGCGC	TGG
OTS13	chr8:4066414-4066392	GCTTCTctGATgACCTGCGg	AGG
OTS14	chr13:78633999-78634021	GCTTCTGtGATTtCCTGgGC	AGG
OTS15	chr7:2524584-2524562	GCgTCTGGGATTctCTGCGC	AGG
OTS16	chr14:103007294-103007316	tCcTCTtGGATTAtCTGCGC	TGG
OTS17	chr12:57693491-57693513	aCTTCTGGaATTcCCTGCcC	TGG
OTS18	chr10:63820891-63820913	GgTTCTGaGATTACCaGCGg	TGG
OTS19	chr1:9483516-9483538	GCTcCTGGGAcTcCCTGCcC	TGG
OTS20	chr13:82698949-82698927	GCTcCTGtGATTACCTGttC	TGG
OTS21	chr14:24324005-24324027	GCTTCTGGGccTcCCTGCcC	AGG
OTS22	chr6:2826090-2826068	GCTTCTGGGgTgtCCTGCtC	GGG
OTS23	chr7:10821380-10821402	tCTTCTGGGAgTACCTGaGa	AGG
OTS24	chr7:122002962-122002984	GCTTCTGGGATTAgCaGaGC	TGG
OTS25	chr15:65568429-65568451	GCTTCTGGGAgTAgCTGgGC	AGG

Disease Models & Mechanisms • Supplementary information

Table S2: Primes used for detection of off-target sites of *ApoE*-sgRNA.

OST	Forward primer (5′-3′)	Reverse primer (5′-3′)	Product (bp)
OTS1	tcccacagccaataacgacc	ggaaaggtaatcgctcgggt	382
OTS2	gcatctaagagaacacccagt	gtgtgggtgtgaaccagtct	252
OTS3	ctcacccttccctccatgag	caaatgtggtctgatgcccc	523
OTS4	cagatcacatcacaccgctg	aacagatggtgaagggacgt	560
OTS5	acggggtgcttactctgttc	gccctacaggtcaacggaaa	389
OTS6	aagaagagccaagggagcat	gatggccttatcctccctcc	624
OTS7	cgcgtgctctgtcatcatta	gaaaccgcccggtgctaaa	302
OTS8	cacagcttctaggtccccaa	cattgctgcacccttgtttt	300
OTS9	ttctttcccaaacccatgcg	ggtccagtgttcctactgct	596
OTS10	gacccctagattcccacgag	gtgctgtggttttaagggca	699
OTS11	gatgccctggtccgaagag	ggggtgaggcgtacagataa	330
OTS12	gggatgaggaaaggaaatgcc	tgtcaaattttaggaggcaagtga	205
OTS13	ccctttgctggaaccctttc	ggctctcaccttctccagtt	497
OTS14	tgagcgtggatggtaataacac	tgggatcgaaggactctgaca	305
OTS15	tatcagttgcaggggagagg	agcatccgaattgtctccca	324
OTS16	attgcttcattaaaaggccacc	acctgaggctgttaaagttgg	330
OTS17	cactgctgacacaagcaactg	tgggatctctggaggggagt	359
OTS18	ctccacccgtgtcctttcaa	ctgacttcacacctgggtcg	302
OTS19	gggtcttggcagaggagag	aatcttttgccgcttctccc	315
OTS20	aaggagctagaagcttgggc	cagcaacactgcgatctgtg	325
OTS21	gggagcgtgctttctattgg	cctccaaggtgaaatgtggc	304
OTS22	acaccaccccttgtctgaaa	ctgtgtcccggctgaaattc	360
OTS23	caggctttcctgtcaaggagt	tgttcaagtgcacgctctcc	304
OTS24	tgtggtctgatcccctcctta	gtgtcaaagccgcagggtg	253
OTS25	gctctgaggacagtcaaggac	cctggtcatttaggcctcct	311

Disease Models & Mechanisms • Supplementary information
